# Pattern separation by neuronal turnover in a feed-forward network

**DOI:** 10.1186/1471-2202-14-S1-P135

**Published:** 2013-07-08

**Authors:** Anthony J DeCostanzo, Tomoki Fukai

**Affiliations:** 1 Brain Science Institute, RIKEN, Wakoshi, Saitama, Japan

## 

Numerous studies have revealed a role for the hippocampal dentate gyrus in behavioral discrimination between similar contexts or objects, referred to as pattern separation. Recently many behavioral studies have demonstrated a role for dentate neurogenesis in such pattern separation. While several computational studies have modeled the effect of neuronal turnover on learning in simple [1-3] and more detailed networks [4], the computational advantage of neurogenesis for pattern separation has remained obscure. Here we present a simple, feed-forward network, with a biologically plausible learning rule for implementing neuronal recycling that reveals the pattern separation properties of neurogenesis. Our model consists of a three-layer network including entorhinal cortical inputs (EC), dentate gyrus (DG) as the hidden layer, and a single CA3 readout unit. The weights between EC and DG are fixed random, while those from DG to CA3 are trained with a perceptron learning rule. A "context" consists of a set of patterns presented to EC with their respective target readouts at CA3. Pereceptron training results in a weight vector with approximately normally distributed weights. Neurogenesis is implemented by replacing units with weak weights to the CA3 readout, thus a new hidden unit with random weights replaces the prior unit. Thereupon another round of perceptron learning is implemented. This simple rule results in a markedly reduced generalization error at the CA3 readout that continues to decline with each subsequent round of neurogenesis. Intuitively, this neurogenesis improves linear separability in the DG space allowing the CA3 readout to draw a decision hyperplane with a greater margin between the classes. This form of neuronal turnover may represent a biologically plausible replacement for error backpropagation.
